# Development and Evaluation of Oro-Mucosal Drug Delivery System for the Effective Management of Oropharyngeal Candidiasis: An Animal Study

**DOI:** 10.7759/cureus.85040

**Published:** 2025-05-29

**Authors:** Prakhar Kapoor, Revati S Deshmukh, Rizwan M Sanadi, Shamolie S Deshmukh

**Affiliations:** 1 Oral and Maxillofacial Pathology, Sinhgad Institute of Dental College, Pune, IND; 2 Oral Pathology and Microbiology, Bharati Vidyapeeth (Deemed to be University) Dental College and Hospital, Pune, IND; 3 Periodontology and Oral Implantology, Dr G. D. Pol Foundation YMT Dental College &amp; Hospital, Navi Mumbai, IND; 4 Dentistry, Bharati Vidyapeeth (Deemed to be University) Dental College and Hospital, Pune, IND

**Keywords:** antifungal, chitosan, fluconazole, nanoparticles, nystatin, oral candidiasis, oropharyngeal candidiasis, posaconazole

## Abstract

Background

Oral candidiasis (OC) is a frequently occurring opportunistic fungal infection seen in individuals with weakened immune systems, such as those with AIDS, those with diabetes mellitus, those undergoing chemotherapy for cancer, or those on prolonged antibiotic medication. Traditional formulations for OC, including mouth paints, rinses, troches, lozenges, and oral gels, cannot sustain the salivary levels of active ingredients over an extended duration. Posaconazole is an extended-spectrum triazole antifungal used to treat invasive candidiasis. A sustained release of this agent is essential for maintaining the salivary concentration for a long. Hence, this study was undertaken to develop a safe and effective posaconazole drug formulation for the management of oropharyngeal candidiasis.

Aim

The aim of this study was to develop a safe and effective drug formulation for the management of oropharyngeal candidiasis.

Methods

The study was conducted in two phases namely: experimental phase I (formulation phase) and experimental phase II. Experimental phase I (formulation phase) consisted of drug and excipient procurement, formulation of chitosan-coated poly-lactic-co-glycolic acid (PLGA) nanoparticles, preparation of chitosan-coated PLGA nanoparticles containing posaconazole, and characterization of formulation. Subsequently, the experimental phase II was carried out, which consisted of conducting in vitro and in vivo studies. In vitro studies consisted of assessing the agar gel diffusion method and time-dependent fungicidal activity. In vivo studies were carried out on animal models (rats) after obtaining ethical clearance from the Animal Ethics Committee.

Results

The in vitro release profile of posaconazole from chitosan/PLGA nanoparticles over 60 h showed that the nanoparticles prolonged the posaconazole release. Nanoformulation was found to be quite active against the *Candida albicans* strain. In candidiasis infection, the results showed an abnormal hematological index. The abnormal hematological indices and the abnormal histopathological changes in candidiasis infection were significantly improved on treatment with nanoformulation. Abnormally increased serum CRP and TNF-α levels were decreased gradually on treatment with the nanoformulation at the end of day 8.

Conclusion

Local treatment of OC with buccal mucoadhesive chitosan-coated nanoparticles of posaconazole was found to be beneficial not only in reducing the overall required dosage and minimizing side effects but also in eliminating the possibility of drug interaction that is encountered during systemic therapy of posaconazole.

## Introduction

Oral candidiasis (OC) is a frequently occurring opportunistic fungal infection seen in individuals with weakened immune system, such as in those with AIDS, those with diabetes mellitus, those undergoing chemotherapy for cancer, or those on prolonged antibiotic medication. OC is primarily caused by *Candida albicans* along with other pathogenic yeasts. Although various antifungal agents have been used to treat OC, the use of high concentrations of these agents can lead to the emergence of resistant strains of *Candida*, as well as potential side effects and drug interactions [1-5].

Nystatin at doses of 100,000 IU/mL (5 mL four times daily) and amphotericin B at 50 mg (5 mL three times per day) have been the first choice for the treatment of candidiasis. Fluconazole suspension in distilled water (2 mg/mL) was used in the treatment of pseudomembranous candidiasis. Itraconazole oral suspension 10 mg/mL dose gave better results than nystatin. The poor adherence of nystatin to oral mucosa and thus the quick ingestion of its suspension resulted in a lower efficiency [6].

Posaconazole is an extended-spectrum triazole with potent in vitro activity against pathogenic yeasts and molds, including fluconazole- and itraconazole-resistant *Candida* strains. In addition, posaconazole treatment has been effective for patients with invasive *Candida glabrata* and *Candida tropicalis* infections who are either intolerant of or have disease refractory to other antifungals [7].

Traditional formulations such as mouthwashes, rinses, troches, lozenges, and oral gels are available for managing OC [8]. However, these formulations are unable to sustain the salivary levels of active ingredients over an extended duration. Mucoadhesive drug delivery systems, which stick to the buccal mucosa and stay in position for an extended duration, are ideal options for treating OC [9-10].

Nanoparticles coated with chitosan have attracted a special interest for mucoadhesive applications, mainly because of their ability to interact with the negatively charged mucosal surface, increased retention time, mucoadhesive properties, and increased local concentration of nanoparticles [11-13]. Chitosan-coated nanoparticles have reached an important position in the arena of drug delivery [14].

Khan et al. [15] employed a single emulsion technique for the formulation and evaluation of forskolin-loaded chitosan-coated poly-lactic-co-glycolic acid (PLGA) nanoparticles and concluded that forskolin-loaded chitosan-coated PLGA nanoparticles can be effectively developed and used as an alternative to traditional methods such as eye drops for managing acute glaucoma.

A mucoadhesive nanoparticle formulation with fluconazole for the treatment of OC was prepared. It showed improved patient compliance due to its extended action. Furthermore, the formulation exhibited no cytotoxic effects at the concentrations that were tested [16]. Hence, the present study was conducted to develop chitosan-coated PLGA nanoparticles of posaconazole and test its efficacy and safety by in vitro studies and animal models for the treatment of oropharyngeal candidiasis (OPC).

## Materials and methods

**Figure 1:**
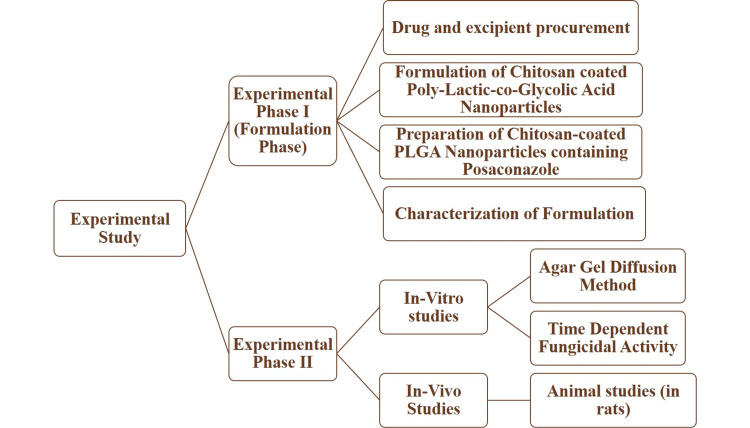
Flowchart of the study PLGA, poly-lactic-co-glycolic acid

The study was conducted in two phases: Experimental phase I (formulation phase) and experimental phase II as described below and in the flowchart of the study (Figure 1). Animal Ethics Committee approval was taken from Sciore Research Private Limited (SC/IAEC/2021/007, dated 24/02/2021).

Experimental phase I (formulation phase)

Drug and Excipient Procurement

Posaconazole was received from Dr. Reddy's Laboratories (Hyderabad, Telangana, India). Polyvinyl alcohol (PVA; Mw: 30,000-70,000; 80-90% hydrolyzed) was procured from Sigma-Aldrich (Bengaluru, Karnataka, India). PLGA was obtained from Evonik Industries. Primex ehf (Siglufjordur, Iceland) provided the chitosan (ChitoClear, medium molecular weight, 96% deacetylated, viscosity 15 cp). A dialysis membrane was purchased from HiMedia Laboratories (Mumbai, Maharashtra, India) (110; average flat width: 31.13 mm, average diameter: 21.5; Mw 12,000-14,000). Ultrapure water was obtained using the Milli-Q Plus System (Millipore, Bengaluru, Karnataka, India). All additional compounds were of the highest purity commercially available and were of analytical grade. Table 1 represents the materials used for the study.

**Table 1:** Materials used in the study

Materials	Manufacturer Details
Posaconazole	Dr. Reddy's Laboratories, Hyderabad, Telangana, India
Polyvinyl alcohol (MW: 30000-70000; 80–90% hydrolyzed)	Sigma–Aldrich, Bengaluru, Karnataka, India
poly (D, L-lactide-co- glycolide; 50:50 (Resomer 504H))	Evonik Industries
Chitosan (ChitoClear, medium molecular weight, 96% deacetylated, viscosity 15 cp)	Primex ehf (Siglufjordur, Iceland)
Dialysis membrane posaconazole (110; average flat width: 31.13 mm, average diameter: 21.5; Mw 12,000-14,000	Himedia Laboratories, Mumbai, Maharashtra, India
Ultrapure water	Milli-Q Plus System (Millipore, Bengaluru, Karnataka, India)

Formulation of Chitosan-Coated Poly-Lactic-co-Glycolic Acid Nanoparticles

Aqueous solution (20 mL total solution) was prepared using chitosan 1% and PVA 1%. The organic solution was prepared using PLGA 50 mg, drug 10 mg, and ethanol 1 mL. Sonication was carried out for 5 minutes by adding organic solution drop by drop into the aqueous phase with a cold jacket. The solution was kept on a magnetic stirrer and stirred at minimum temperature to evaporate organic phase and later cold centrifuged at 4°C for 15 minutes to separate the supernatant.

Preparation of Chitosan-Coated PLGA Nanoparticles Containing Posaconazole

Posaconazole-containing PLGA nanoparticles coated in chitosan were created using a modified version of the emulsion solvent evaporation method, as previously described by Carraro et al. [17]. In the organic phase, PLGA and posaconazole were divided in the ratio of 50:50 and dissolved in 1 mL of dichloromethane.

The aqueous phase included 0.5% chitosan and 2% PVA that were dissolved in a solution of 1% acetic acid with a pH of 5.0. A probe sonicator (60 W, 35% duty cycle, Hielscher Ultrasonics, Berlin, Germany) was used to combine and emulsify the two phases. The mixture was then left on an ice bath for 3 minutes. The mixture was left on a magnetic stirrer at 1,000 rpm for 6 hours at room temperature to allow the solvent to completely evaporate. To eliminate any extra stabilizer, the formulated mixture was centrifuged using a TOMY Mx-305 High-Speed Refrigerated Microcentrifuge at 15,000 rpm for 30 minutes at a temperature of 4°C.

The produced pellet was redispersed in Milli Q water after being combined with mannitol (2%), which serves as a cryoprotectant. The pellet was lyophilized in a lyophilizer (Labconco Lyph Lock 6 Floor Model Freeze Dryer, East Lyme, CT, USA) for 24 hours to produce freeze-dried nanoparticles after the pellet had been freeze-dried for 10 to 12 hours. Phosphate-buffered saline (PBS) was used to redisperse the lyophilized nanoparticles (pH 7.4).

Characterization of Formulation

Particle size and morphology: Using the dynamic light scattering technique, particle size was estimated. DTS (nano) software was used in conjunction with photon correlation spectroscopy (Zetasizer, HAS.3000, Malvern Instruments, Malvern, UK). At 25°C and a 90-degree detection angle, the analysis was continued. Malvern Zetasizer (Nano ZS-90) was used to conduct zeta potential analysis on the nanoparticles to ascertain their surface charge and aggregation behavior.

Differential scanning calorimetry (DSC): Using DSC, the thermal behavior of the formulation materials, PLGA and chitosan-coated PLGA nanoparticles, was examined. To guarantee the correctness and precision of the obtained DSC thermograms, calibration with indium was carried out. T zero aluminum pans with a pinhole lid were loaded with precisely weighed samples weighing 5-10 mg. Under a nitrogen environment, the analysis was conducted up to 3500°C at a heating rate of 10°C/minute.

Entrapment efficiency: A microcentrifuge (TOMY, MX-305, High Speed chilled) set at 15,000 rpm for 30 minutes at 4°C was utilized to separate the nanoparticles from an aqueous suspension (entrapment). The effectiveness of posaconazole-loaded chitosan-coated PLGA NPs was evaluated using a high-performance thin-layer chromatography analysis. The following equation and the amount of medication in the clear supernatant was determined:

EE (%) = Total amount of drug - Amount of drug in supernatant x 100 / Weight of nanoparticles

where EE stands for entrapment efficiency

In vitro drug release: In a pre-treated dialysis bag, release studies were conducted using 2 mL of chitosan-coated PLGA nanoparticles submerged in 50 mL of the dissolving medium. Simulated tear fluid ([STF] pH 7.4) and methanol were combined in this medium in a 9:1 ratio. NaCl 0.68 g, KCl 0.14 g, NaHCO3 0.22 g, and CaCl2×2H2O 0.008 g were combined to form STF in 100 mL of Milli Q water. At 37° ± 1°C, the dissolving process was maintained while being stirred at 150 rpm.

For the first 72 hours, aliquots from the release media were removed for drug content analysis and reintroduced with the same quantity at predetermined intervals. The following equation was used to compute the percentage of medication release:

Drug release (%) = (Released drug / Total drug) x 100

Based on regression coefficient (R2) values, the mathematical models zero and first order, Hixson-Crowell, Higuchi, and Korsmeyer-Peppas were used to analyze the best curve fit of the in vitro drug release [18].

Experimental phase II

In Vitro Studies

Agar well diffusion method: The anticandidal effects of selected optimal formulations were assessed using the agar well diffusion method. Fresh cultures of *C. albicans* were adjusted to a 0.5 McFarland turbidity in a sterile physiological saline solution and spread onto Mueller-Hinton Agar (G-MB-MHA) plates supplemented with glucose and methylene blue and then allowed to dry. Subsequently, 1 mL of 0.5% of PLGA-coated nanoparticle formulation was aseptically introduced into 12-mm-diameter wells drilled into the inoculated glucose- and methylene blue-added Mueller-Hinton Agar medium, after which the plates were incubated at 35°C. After 24 to 48 hours, the diameters of the inhibition zones were measured. The experiments were repeated three times, and the average measurements were calculated. For assessing possible contamination, the preparations were similarly processed on agar plates without *C. albicans*.

Time-dependent fungicidal activity: The formulated mixtures were combined with an equal volume of a 0.5 McFarland adjusted suspension of *C. albicans* in sterile PBS within test tubes. The combined samples were incubated at a temperature of 35°C. At various time points, samples from these mixtures were taken and diluted in physiological serum at ratios of 1:10 and 1:100. The diluted samples were then inoculated onto Sabouraud Dextrose Agar (SDA) plates and incubated for a period of 24 to 48 hours. The viability of the cells was determined by counting the number of yeast colonies that developed on the SDA plates. The blank formulations were evaluated as negative controls, while the PBS-*C. albicans* mixture was used as a growth control.

In Vivo Studies

OPC inducing model: Animal Ethics Committee approval was taken from Sciore Research Private Limited (SC/IAEC/2021/007 dated 24/02/2021). Rats were purchased. To induce OC, 50 μL of *C. albicans* suspensions with 106 CFU/mL of microorganisms in PBS was administered to the oral mucosa of the rats daily using cotton swabs for a duration of six days. To verify the presence of OC, oral mucosal smears were collected daily starting on the third application, and these smears were inoculated onto an SDA medium. After incubating at 35°C for 48 hours, the growth of *C. albicans *was evaluated between 24 and 48 hours.

The animals that tested positive for infection were categorized into three distinct groups: the first group received no treatment, the second group was administered a dispersion of chitosan-coated PLGA nanoparticles combined with posaconazole, and the third group was treated with a commercially available OC paint (clotrimazole).

Oral mucosal smears were collected from the animals on days 1 through 7 of the treatment and inoculated into an SDA medium. Following incubation at 35°C for 48 hours, *C. albicans* growth was established at the 24- to 48-hour mark, and the effectiveness of the formulations was examined.

Following the full course of the infection and the subsequent healing of the animals, the rats were euthanized with a dose of 150 mg/kg of pentobarbital. The buccal, palatal, and tongue mucosa from the animals were then removed and preserved in 10% formalin for the standard tissue processing needed for histological analysis using light microscopy.

Inclusion Criteria

The most common locations for OC in animals are the buccal mucosa, palate, and tongue, which were utilized as the test areas.

Exclusion Criteria

Prior to introducing Candidal swab into the oral cavity, samples from the buccal mucosa, palate, and tongue of the animals were inoculated on an SDA medium to control yeast colonization. Rats that were already infected with yeast were excluded from the study.

Statistical analysis

All data were entered into a computer by giving a coding system and proofed for entry errors. Data obtained were compiled on an MS Office Excel Sheet (v 2019, Microsoft Corp., Redmond, WA, United States). Data were subjected to statistical analysis using the Statistical Package for Social Sciences (v 26.0, IBM Corp., Armonk, NY). Descriptive statistics, namely mean and standard deviation (SD), for numerical data were calculated. The normality of numerical data was checked using the Shapiro-Wilk test, and it was found that the data did follow a normal curve; hence, the parametric test two-way ANOVA was used.

For all the statistical tests, p<0.05 was considered to be statistically significant, keeping α error at 5% and β error at 20%, thus giving power to the study as 80%.

## Results

Measurement of particle size, poly-dispersity index, and zeta potential

In the present study, nanoparticles and coated nanoparticles were successfully prepared adapting the spontaneous emulsification technique. Nanoparticles presented an average diameter lower than 300 nm, poly-dispersity index lower than 0.3, and a positive zeta potential.

Microscopic analysis of posaconazole

Morphology of posaconazole nanoparticles was evaluated, and the microscopic image revealed crystal form. The crystalline form of posaconazole consisted of a large amount of fraction of bigger and rod-shaped crystals.

In vitro release kinetics of chitosan-coated poly-lactic-co-glycolic acid nanoparticles containing posaconazole

The in vitro release profile of posaconazole from chitosan-coated PLGA nanoparticles over 60 hours showed the prolonged release of the drug. A biphasic release profile was obtained, characterized by an initial fast phase, releasing around 41.29% of posaconazole in 6 hours, followed by a much-sustained phase, releasing around 81.63% of posaconazole after 60 hours.

Calibration curve for posaconazole

Linearity of the calibration curve was tested over the range of 10-300 µg-1. To determine the relationship between the absorbance and concentration, optical densities were measured in solutions with growing concentrations of posaconazole. Measurements were carried out in the concentration range of 710-300 µg-1 with potentials from 0 V to 0.8 V.

Spectrophotometric determination

Posaconazole was analyzed spectrophotometrically by the appropriate method to determine the wavelength at maximum absorption (λmax). Absorption spectrum for posaconazole by using UV-visible spectrophotometer was recorded in the range (260 nm) by using quartz cells (Cuvette) of 1 cm thickness (path length).

Differential scanning calorimetric study

To examine the physical state of the drug and the molecular dispersion in the nanoparticles, DSC analysis was performed. PLGA displayed an endothermic peak at 370°C corresponding to glass transition/relaxation and degradation, respectively. Chitosan displayed two endothermic peaks, one at 110°C corresponding to degradation and the other peak at 300°C associated with the melting temperature. The endothermic glass transition/relaxation peak and melting peak of PLGA and chitosan were combined and shifted to 169.058°C and 304.409°C, respectively, for nanoparticle formulations, which suggests the adsorption of chitosan onto PLGA in the nanoparticles. Posaconazole exhibited a sharp endothermic peak at 169.033°C due to the melting point of the crystalline state. The endothermic peaks of the drug and PLGA, occurring at 169.033°C and 370°C, respectively, indicated that the intensity of the drug peak reduced in the physical mixture, possibly because of the dilution effect caused by the polymer. Nevertheless, the degradation peak of PLGA was absent in the formulations, which was attributed to its improved thermal stability.

**Figure 2:**
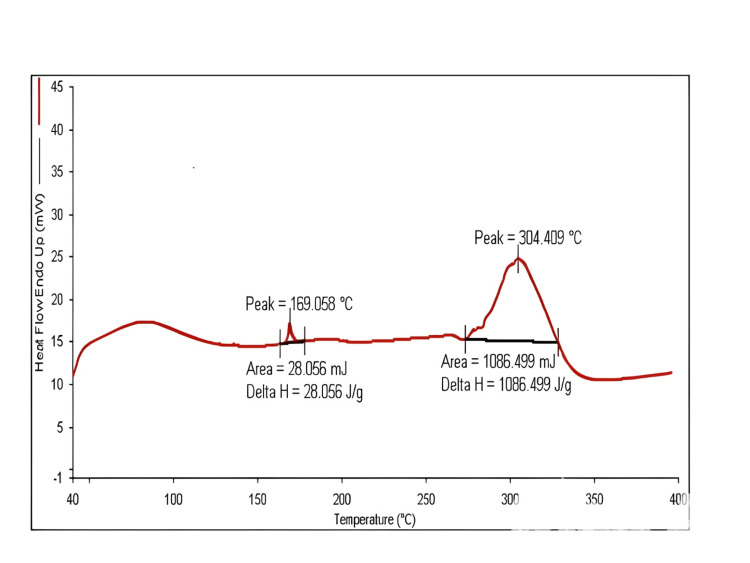
DSC thermogram of physical mixture Peak 1: The endothermic glass transition/relaxation peak and melting peak of poly-lactic-co-glycolic acid was 169.058°C for nanoparticle formulations Peak 2: The endothermic glass transition/relaxation peak and melting peak of chitosan was 304.409°C for nanoparticle formulations DSC, differential scanning calorimetry

The pronounced endothermic peak of the drug was absent in the physical mixture, suggesting a transformation of the drug into an amorphous state that contributes to improved aqueous solubility and molecular distribution within the nanoparticle system. The fusion of endothermic peaks of PLGA and chitosan highlights the entanglement of PLGA chains and chitosan moieties indicating the formation of intact nanoparticles (Figure 2).

Fourier-transform infrared spectroscopic analysis

**Figure 3:**
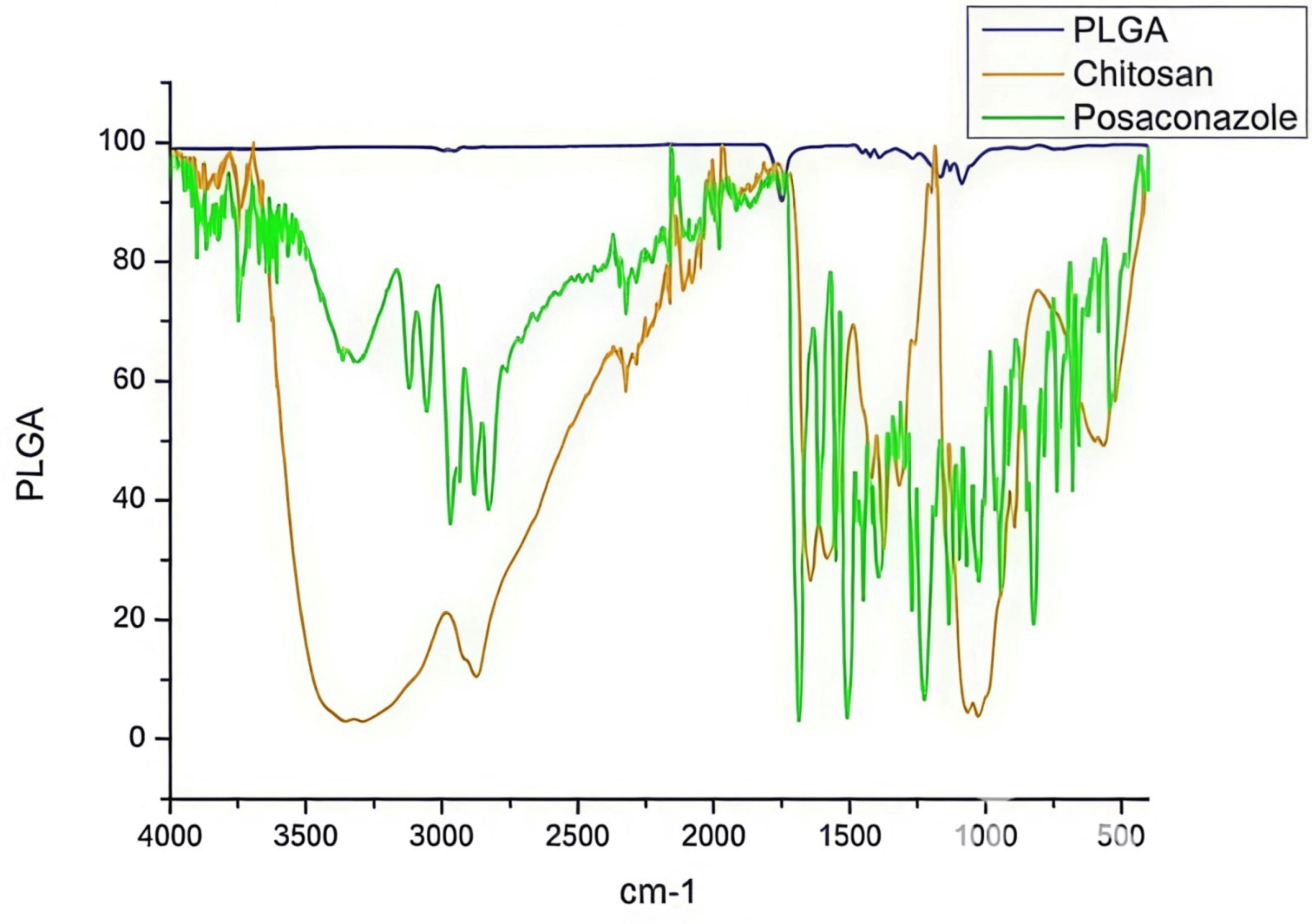
FTIR spectroscopic spectrum analysis of chitosan PLGA nanoparticles containing posaconazole FTIR, Fourier-transform infrared; PLGA, poly-lactic-co-glycolic acid

The Fourier-transform infrared spectra suggested the existence of chitosan coating on PLGA nanoparticles by the presence of characteristics functional group of chitosan showing a slight shift in wave number. The Fourier-transform infrared characteristic peaks of chitosan together with the conversion of negative zeta potential to positive zeta potential and the thermogram changes of coated nanoparticles suggest the successful coating of chitosan onto PLGA nanoparticles. The characteristic negative charge of the structures can be utilized to enhance drug bioavailability by introducing an oppositely charged polymer for surface coating (Figure 3).

In vitro antifungal activity

Nanoformulations were selected for in vitro anti-fungal activity assay studies because of appropriate particle size, poly-dispersity index, and zeta potential.

Agar well diffusion method

**Figure 4:**
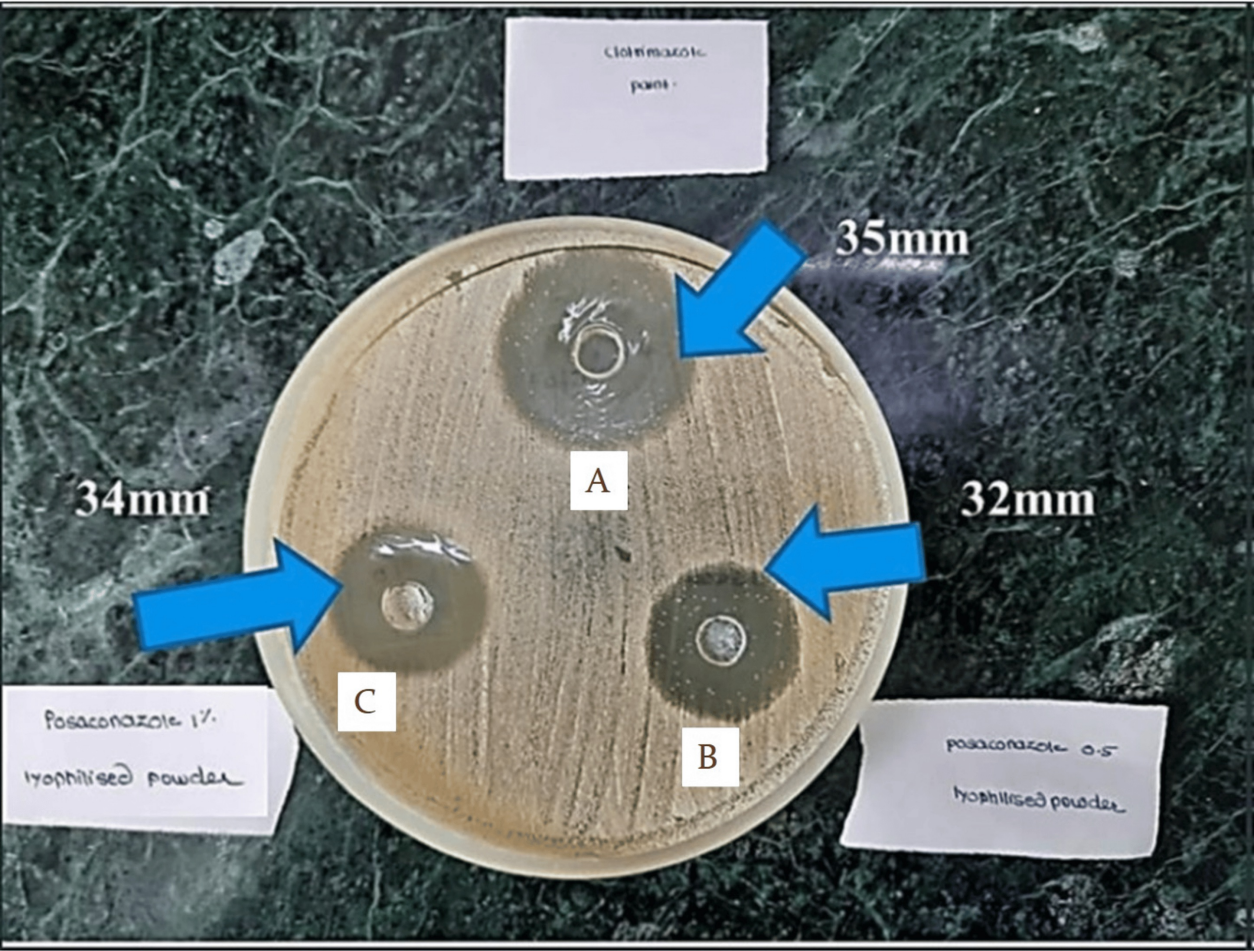
Zone of inhibition caused by the 0.5% and 1% posaconazole formulation on G-MB-MHA medium in comparison to 1% Candid mouth paint A. Zone of inhibition caused by 1% Candid mouth paint on G-MB-MHA medium. B. Zone of inhibition caused by 0.5% posaconazole formulation on G-MB-MHA medium. C. Zone of inhibition caused by 1% posaconazole formulation on G-MB-MHA medium. G-MB-MHA, glucose- and methylene blue-added Mueller-Hinton Agar

The inhibition zone diameters for the nanoformulation were found to be 32 mm and 34 mm for 0.5% and 1% of the drug concentration, respectively. Nanoformulation was found to be quite active against the *C. albicans* strain (Figure 4).

Time-dependent fungicidal activity

**Figure 5:**
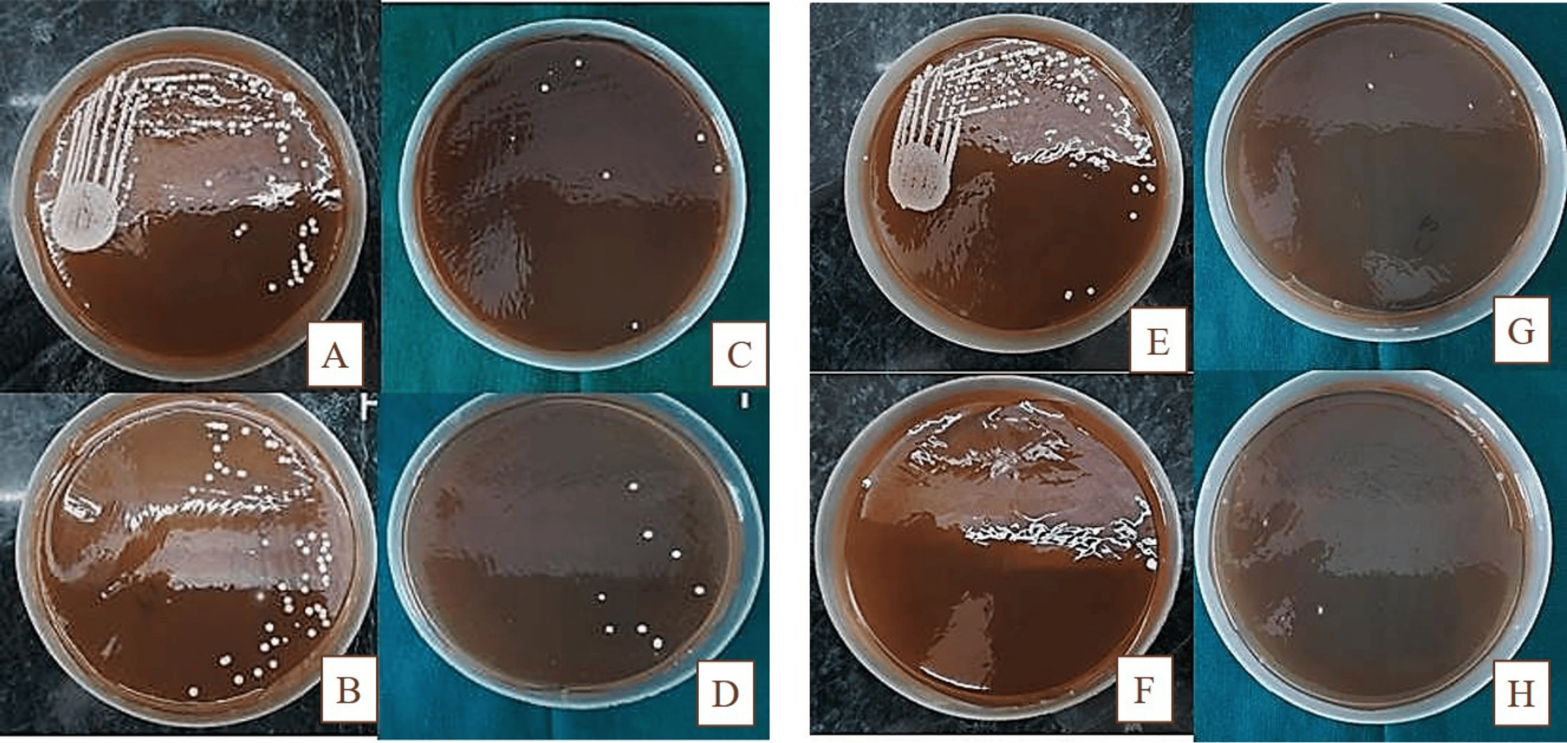
Time-dependent fungicidal activity of candid mouth paint and posaconazole on Sabouraud Dextrose Agar medium A. At baseline after application of Candidal colonies. B. At 0 minutes of application of Candid mouth paint. C. At 30 minutes of application of Candid mouth paint. D. At 60 minutes of application of Candid mouth paint. E. At baseline after application of Candidal colonies. F. At 0 minutes of application of posaconazole nanoparticle formulation. G. At 30 minutes of application of posaconazole nanoparticle formulation. H. At 60 minutes of application of posaconazole nanoparticle formulation.

The prepared formulation reduced the number of cells sharply in a short time. After an hour, the formulation killed all *C. albicans* cells in the suspension in comparison to a few viable colonies still present for 1% Candid mouth paint (Figure 5).

In vivo study

Body Weight

Changes in body weight were recorded on day 0, day 7, day 14, and day 21. Two-way ANOVA revealed that the change in body weight was not significant on day 0 and day 7 of treatments.

Organ Weight

At the end of the study, animals were sacrificed and organs were dissected. There was no significant change observed in relative organ weights of the normal control group when compared to the disease control group.

Food Consumption

Food consumption or food intake was recorded on day 0, day 7, day 14, and day 21. Two-way ANOVA revealed that the food consumption in the disease control group and the test group was decreased significantly on day 7 (P<0.001) in comparison with the normal control group.

Microbial Analysis of Oral Swabs

**Figure 6:**
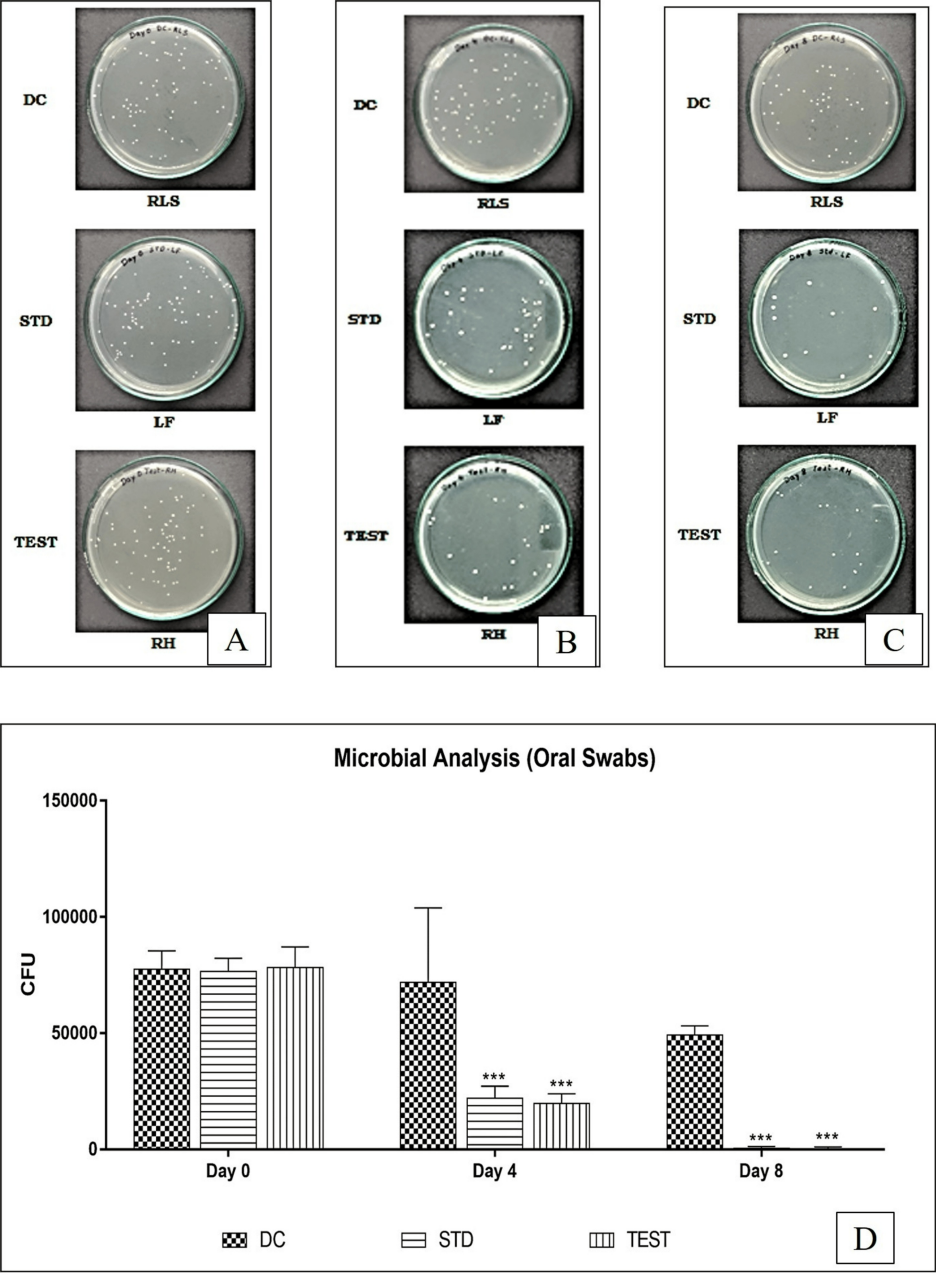
Microbial colony count analysis of oral swabs A. At day 0. B. At day 4. C. At day 8. D. Graphical representation of statistical analysis of the results of two-way ANOVA. ***Statistically very highly significant difference (p<0.001). Test group (posaconazole) was compared with the disease control group and standard group (Candid mouth paint) for statistical analysis. The microbial growth was significantly decreased in the test group as compared to the disease control group and the standard group on day 4 and day 8 (P<0.001). RLS, right leg side; RH, right hind limb; LF, left front limb; LS, left side (for animal identification); DC, disease control (rats with candidiasis); STD, standard (rats treated with Candid mouth paint); TEST, posaconazole formulation (rats treated with posaconazole formulation)

Oral swabs were collected on day 0, day 4, and day 8 during treatment for the evaluation of Candidal growth. The microbial growth was significantly decreased in the standard group and the test group on day 4 and day 8 (P<0.001). Animals were randomized on the basis of microbial analysis of day 0 (Figure 6).

Hematology

At the end of the study, hematology was performed. Significant changes were observed in the levels of white blood cells (WBCs), hemoglobin, platelets, platelet distribution width, procalcitonin, lymphocytes, and neutrophils. It was observed that WBC count, lymphocytes, unclassified WBC count, and neutrophil levels were significantly decreased in the disease control group (P<0.001) in comparison with the normal control group, while the levels are restored in the standard and test groups in comparison with the disease control (P<0.001). Levels of hemoglobin were reduced significantly in the disease control group in comparison with the normal control group (P<0.001), while the levels were restored in the test group (P<0.05).

Serum C-reactive protein, interleukin-6, and tumor necrosis factor-alpha were analyzed using the enzyme enzyme-linked immunosorbant assay (ELISA) method.

Serum C-Reactive Protein

**Figure 7:**
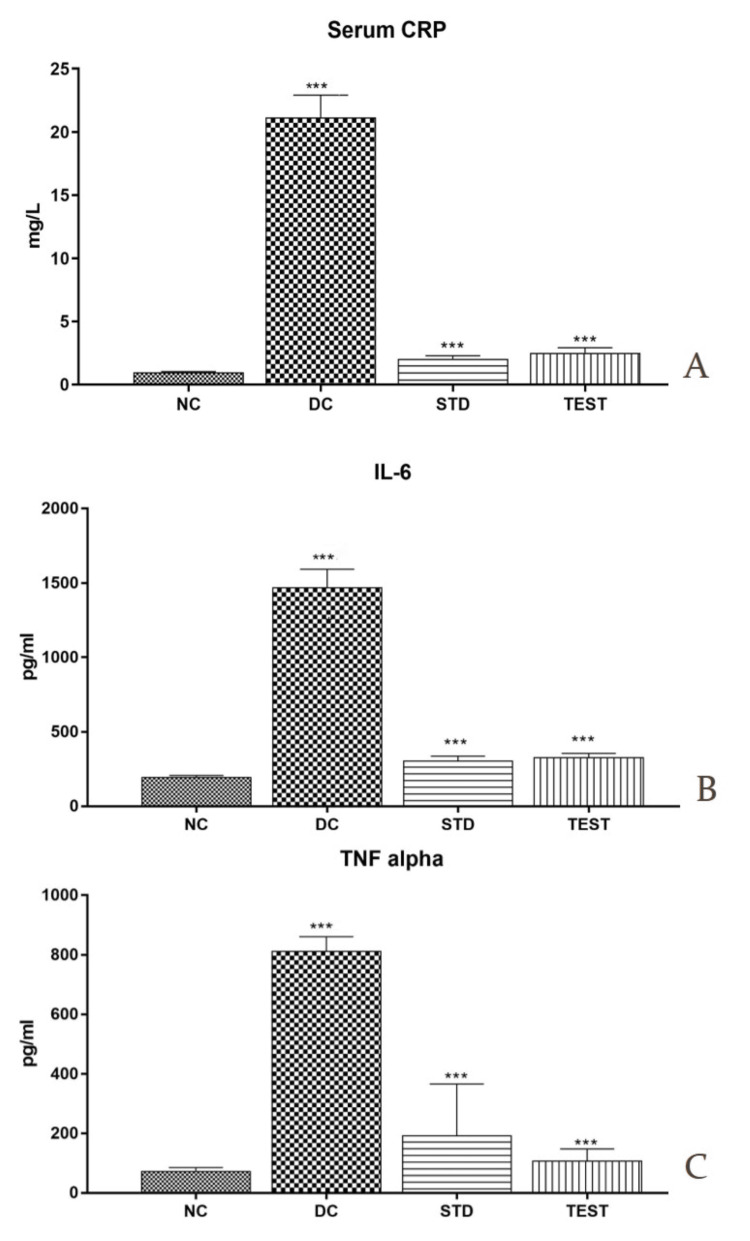
Marker assays serum CRP, IL-6, and TNF-α of normal control, disease control, standard, and test A. Graphical representation of serum CRP levels. B. Graphical representation of IL-6 levels. C. Graphical representation of TNF-α levels. ***Statistically very highly significant difference (p<0.001) Two-way ANOVA test was used for statistical analysis The levels of serum CRP, IL-6, and TNF-α were compared in the normal control group and the disease control group with the standard group and the test group. The levels of serum CRP, IL-6, and TNF-α were significantly increased in the disease control group in comparison with the normal control group, while the levels in the standard group and the test group were decreased significantly in comparison with the disease control group. CRP, C-reactive protein; IL-6, interleukin-6; TNF-α, tumor necrosis factor-alpha; NC, normal control; DC, disease control; STD, standard

Serum levels of C-reactive protein were significantly increased in the disease control group in comparison with the normal control group, while the levels were restored to normal significantly in comparison with the disease control group (P<0.001) (Figure 7A).

Interleukin-6

Interleukin-6 was performed from tissue homogenate. Levels of interleukin-6 were significantly increased in the disease control group in comparison with the normal control group, while the levels were decreased significantly in comparison with the disease control group (P<0.001) (Figure 7B).

Tumor Necrosis Factor-Alpha

Tumor necrosis factor-alpha was performed from tissue homogenate. Levels of the tumor necrosis factor-alpha were significantly increased in the disease control group in comparison with the normal control group, while the levels were decreased significantly in comparison with the disease control group (P<0.001) (Figure 7C).

Histopathology of Tissues

**Figure 8:**
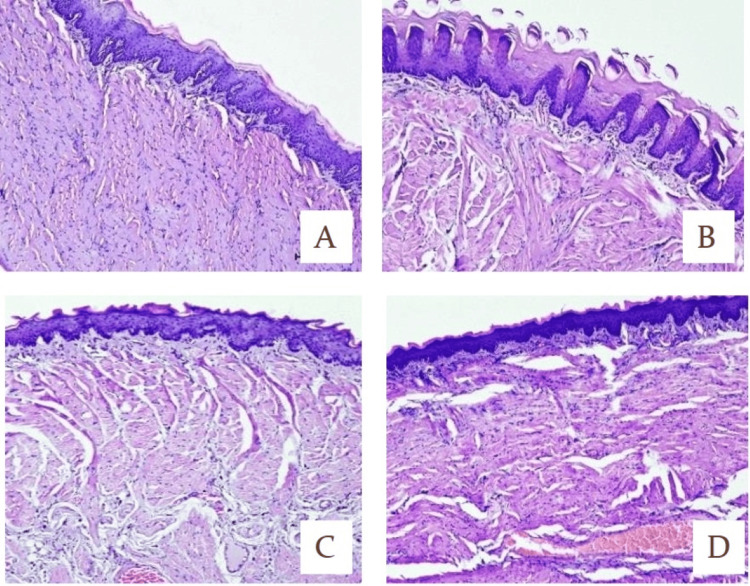
Histopathological images A. Normal control: normal histomorphological features of mucosa and submucosa of the tongue. Absence of any inflammatory or pathological lesion in the tongue tissue sections. B. Disease control: hyperkeratinization of stratified squamous epithelium of the mucosa of the tongue, mild degenerative changes in the mucosal epithelium tissue with loss of mucosa occasionally with mild infiltration of mononuclear inflammatory cells. C. Standard formulation (Candid mouth paint): normal histomorphological features of mucosa and submucosa of the tongue, congested vascular tissue focally, focal infiltration of mononuclear inflammatory cells, absence of any inflammatory or pathological lesion in the tongue tissue sections. D. Test (posaconazole nanoparticle formulation): normal histomorphological features of mucosa and submucosa of tongue, congested vascular tissue focally, focal infiltration of mononuclear inflammatory cells, absence of any inflammatory or pathological lesion in the tongue tissue sections.

Histopathological observations were done. In candidiasis, sections showed predominantly spongiotic changes in the epidermis with irregular acanthosis, mild spongiosis, and inflammatory changes. In the superficial epidermis, the characteristic feature is the presence of neutrophils in the stratum corneum and upper layers of the epidermis. The neutrophils may form small collections (spongiform postulation), which resemble impetigo or psoriasis. The abnormal histopathological changes in candidiasis infection were significantly improved on treatment with nanoformulation (Figure 8).

## Discussion

Candidiasis is the most prevalent fungal illness that affects the mucosa, skin, nails, and internal organs in humans. Candidiasis is caused by various kinds of yeast-like fungi in the genus *Candida*. The infection can be acute or chronic, superficial or deep, and its clinical spectrum is broad. It is most commonly diagnosed as a secondary/superimposed infection in people with immuno-compromised conditions and only rarely as the primary disease [19].

*Candida albicans* is the species of *Candida* most frequently linked to OPC. *Candida albicans* is isolated in approximately 70% of patients with human immunodeficiency virus (HIV) positivity. *Candida glabrata*, *Candida tropicalis*, *Candida parapsilosis*, or *Candida dublinensis* are discovered less frequently [20-22]. OPC is frequent in HIV-positive individuals with CD4 counts under 200 cells/L, chemotherapy-treated immuno-compromised individuals, individuals taking systemic steroids, and individuals using inhaled steroids. OPC develops in 80% to 90% of AIDS patients [23].

Posaconazole, the most recent azole antifungal to receive FDA approval, has a broad spectrum of efficacy against a variety of yeasts and filamentous fungi. Although it can be obtained as an oral suspension and is typically well tolerated by patients, gastrointestinal absorption is sometimes inadequate and remains a challenge in the therapy of persistent infections [24]. OPC, including the azole-resistant condition, can be efficiently treated with it. It is frequently utilized and successful for the prevention of invasive fungal infections in individuals with weakened immune systems [21,23].

Posaconazole is a triazole antifungal medication that resembles itraconazole structurally. By blocking the enzyme lanosterol 14-alpha demethylase, posaconazole prevents the formation of ergosterol, a sterol that is a component of fungal cell membranes. This results in an accumulation of methylated sterol precursors [25]. Posaconazole is not significantly metabolized by the CYP450 enzymes, unlike other azole antifungals [26]. Posaconazole has been demonstrated to be effective against a variety of filamentous fungus in in vitro experiments, including *Candida *species, *Aspergillus *species, *Coccidioides *species, *Fusarium *species, *Histoplasma capsulatum*, Zygomycetes, and Phaeohyphomycetes [27].

Posaconazole has also been effective against *Candida* species that are resistant to fluconazole and itraconazole, as well as Aspergillus fumigatus that is resistant to itraconazole, voriconazole, and amphotericin B. Additionally, it has shown effectiveness against *Cryptococcus neoformans* [27]. Posaconazole is fungicidal to some Candida species (*Candida lusitaniae*, *Candida krusei*, *Candida kefyr*, and *Candida inconspicua*), but fungistatic to others (*Candida albicans*, *Candida glabrata*, *Candida parapsilosis*, and *Candida tropicalis*) [28].

Posaconazole has been given FDA approval for a number of clinical uses, including the prevention of invasive aspergillosis in high-risk patients, the prevention of disseminated candidiasis in severely immuno-compromised patients, and the treatment of OPC, including OPC that is resistant to itraconazole and/or fluconazole. Clinical applications such as invasive aspergillosis or esophageal candidiasis are not FDA-approved [29].

Posaconazole works effectively for treating some fungus, including Aspergillus and some Zygomycetes, as well as for preventing invasive fungal infections in a subset of patient populations. For the prevention of invasive fungal infections in patients with neutropenia from chemotherapy, leukemia, or myelodysplastic syndrome, posaconazole has been demonstrated to be more effective than fluconazole or itraconazole. Posaconazole is at least equally efficacious as fluconazole in treating OPC in HIV patients with OPC. Posaconazole works well for treating fluconazole/itraconazole-resistant OPC infections as well as fluconazole-resistant *Candida* species infections in non-OPC species. Posaconazole side effects are typically comparable to those of fluconazole in trials; however, administration and absorption are still very problematic [30].

Based on limits in the formulary, some insurance companies or organizations could need prior authorization. Posaconazole suspension may also only be available right away in selected hospitals, clinics, and neighborhood pharmacies, depending on the patient populations in the area. In general, patients should be informed about the goal of treatment, administration instructions, and potential side effects of posaconazole therapy to increase patient compliance with OPC posaconazole therapy.

## Conclusions

Drug-loaded nanoparticles can achieve greater drug encapsulation efficiency (up to 100%) and cellular absorption, resulting in greater therapeutic effects. The current study seeks to encapsulate posaconazole in PLGA nanoparticles capped with chitosan to increase antifungal activity and release behavior when compared to free posaconazole.

The current study highlights the need to create dose forms in such a way that the drug is released in a regulated manner, reducing intra-patient and inter-patient variability. Posaconazole improved absorption and decreased inter-subject variability may be attributed to the bioadhesive polymer. Posaconazole, the drug utilized in the study, can have its variability decreased by changing its release pattern. Local treatment of OC with buccal mucoadhesive chitosan-coated nanoparticles would be beneficial not only to reduce the overall required dosage and minimize side effects but also to eliminate the possibility of drug interaction that is encountered during systemic therapy of posaconazole. During the course of the trial, no serious adverse events were reported. Overall, there were no notable differences or trends in laboratory results between screening and final examination. All of the individual rat's vital signs were satisfactory during the trial. At the given dose, the formulations were safe and well tolerated.
